# GlobalFungi, a global database of fungal occurrences from high-throughput-sequencing metabarcoding studies

**DOI:** 10.1038/s41597-020-0567-7

**Published:** 2020-07-13

**Authors:** Tomáš Větrovský, Daniel Morais, Petr Kohout, Clémentine Lepinay, Camelia Algora, Sandra Awokunle Hollá, Barbara Doreen Bahnmann, Květa Bílohnědá, Vendula Brabcová, Federica D’Alò, Zander Rainier Human, Mayuko Jomura, Miroslav Kolařík, Jana Kvasničková, Salvador Lladó, Rubén López-Mondéjar, Tijana Martinović, Tereza Mašínová, Lenka Meszárošová, Lenka Michalčíková, Tereza Michalová, Sunil Mundra, Diana Navrátilová, Iñaki Odriozola, Sarah Piché-Choquette, Martina Štursová, Karel Švec, Vojtěch Tláskal, Michaela Urbanová, Lukáš Vlk, Jana Voříšková, Lucia Žifčáková, Petr Baldrian

**Affiliations:** 1 Institute of Microbiology of the Czech Academy of Sciences, Vídeňská 1083, 14220 Praha 4, Czech Republic; 2 Laboratory of Systematic Botany and Mycology, University of Tuscia, Largo dell’Università snc, Viterbo, 01100 Italy; 3 Department of Forest Science and Resources, College of Bioresource Sciences, Nihon University, Fujisawa, Kanagawa Japan; 4 Department of Biology, United Arab Emirates University, Al Ain, Abu Dhabi, United Arab Emirates; 5 Section for Genetics and Evolutionary Biology, University of Oslo, Blindernveien 31, 0316 Oslo, Norway

**Keywords:** Fungal ecology, Biogeography

## Abstract

Fungi are key players in vital ecosystem services, spanning carbon cycling, decomposition, symbiotic associations with cultivated and wild plants and pathogenicity. The high importance of fungi in ecosystem processes contrasts with the incompleteness of our understanding of the patterns of fungal biogeography and the environmental factors that drive those patterns. To reduce this gap of knowledge, we collected and validated data published on the composition of soil fungal communities in terrestrial environments including soil and plant-associated habitats and made them publicly accessible through a user interface at https://globalfungi.com. The GlobalFungi database contains over 600 million observations of fungal sequences across > 17 000 samples with geographical locations and additional metadata contained in 178 original studies with millions of unique nucleotide sequences (sequence variants) of the fungal internal transcribed spacers (ITS) 1 and 2 representing fungal species and genera. The study represents the most comprehensive atlas of global fungal distribution, and it is framed in such a way that third-party data addition is possible.

## Abstract (summary)

**Table:** 

Measurement(s)	internal_transcribed_spacer_region
Technology Type(s)	digital curation
Factor Type(s)	geographic location • sample type • biome • sampling year
Sample Characteristic - Organism	Fungi
Sample Characteristic - Environment	terrestrial biome

Machine-accessible metadata file describing the reported data: 10.6084/m9.figshare.12481946

## Background & Summary

Fungi play fundamental roles in the ecosystem processes across all terrestrial biomes. As plant symbionts, pathogens or major decomposers of organic matter they substantially influence plant primary production, carbon mineralization and sequestration, and act as crucial regulators of the soil carbon balance^1,2^. The activities of fungal communities contribute to the production of clean water, food, and air and the suppression of disease-causing soil organisms. Soil fungal biodiversity is thus increasingly recognized to provide services critical to food safety and human health^3^.

It is of high importance to determine how environmental factors affect the diversity and distribution of fungal communities. So far, only a few studies have focused on fungal distribution and diversity on global scale^4–6^. Importantly, these single survey studies focused either on a limited number of biomes^4,5^, fairly narrow groups within the fungal kingdom^6^, or were restricted only to fungi inhabiting soil. Although individual studies have the advantage of standardized methodology across their whole dataset, their limitation is in the limited sampling efforts in space and time that do not allow general conclusions on distribution of fungal taxa. On the other hand, since the advent of high-throughput-sequencing methods, large amounts of sequencing data on fungi from terrestrial environments accumulated along with metadata across numerous studies and allow interesting analyses when combined^7^. As an example of this approach, the meta-analysis of 36 papers made it possible to map global diversity of soil fungi collected in >3000 samples and indicated that climate is an important factor for the global distribution of soil fungi^8^. This approach clearly demonstrated the utility of a meta-study approach to address fungal biogeography, ecology and diversity. In addition, the compilation of these data demonstrated the fact that symbiotic mycorrhizal fungi that aid cultivated and wild plants to access nutrients, are more likely to be affected by rapid changes of climate than other guilds of fungi, including plant pathogens^8^ and helped to identify which fungi tend to follow alien plants invading new environments^9^.

**Fig. 1:**
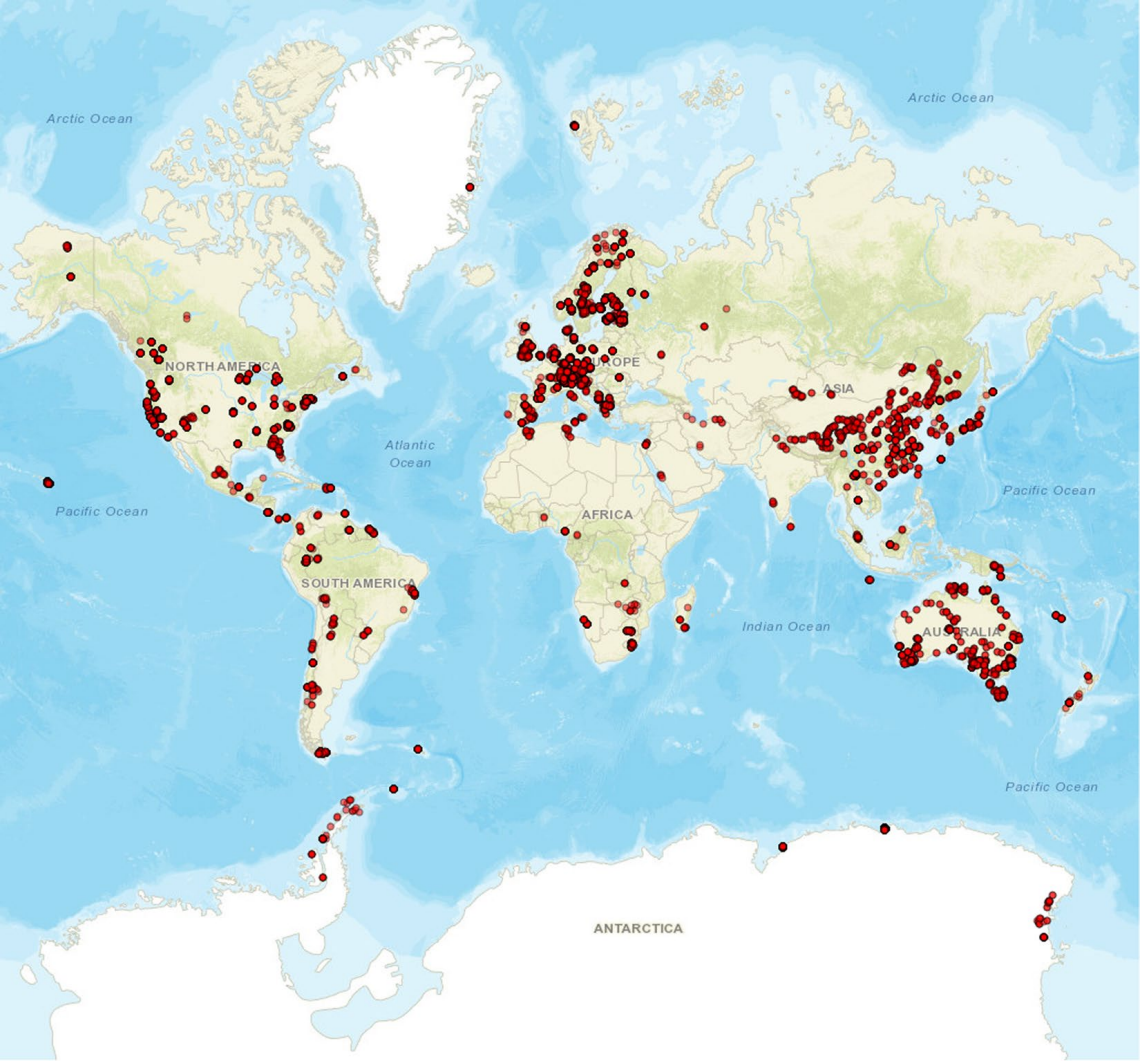
Map of locations of samples contained in the GlobalFungi database. Each point represents one or several samples where fungal community composition was reported using high-throughput-sequencing methods targeting the ITS1 or ITS2 marker of fungi. The map was created using the ‘leaflet’ package that uses an open-source JavaScript library for mobile-friendly interactive maps (Leaflet 1.6.0, GNU General Public License).

Here, we have undertaken a comprehensive collection and validation of data published on the composition of fungal communities in terrestrial environments including soil and plant-associated habitats. This approach enabled us to construct the GlobalFungi database containing, on March 16, 2020, over 110 million unique sequence variants^10^ (i.e., unique nucleotide sequences) of the fungal nuclear ribosomal internal transcribed spacers (ITS) 1 and 2, covering > 17 000 samples contained in 178 original studies (Fig. 1). The ITS region has been used as molecular marker because it is a universal barcode for fungi^11^.The dataset of sequence variant frequencies across samples, accompanied by metadata retrieved from published papers and in global climate databases is made publicly available at https://globalfungi.com. To achieve the goal to make published data findable, accessible, interoperable and reusable, the user interface at the above address allows the users to search for individual sequences, fungal species hypotheses^12^, species or genera, to get a visual representation of their distribution in the environment and to access and download sequence data and metadata. In addition, the user interface also allows authors to submit data from studies not yet covered and in this way to help to build the resource for the community of researchers in systematics, biogeography, and ecology of fungi.

## Methods

### Data selection

We explored papers fitting with a main criterion, i.e., high-throughput sequencing for the analysis of fungal communities thanks to the ITS region, and that were published up to the beginning of 2019; in total, we explored 843 papers. The following selection criteria were used for the inclusion of samples (and, consequently, studies) into the dataset: (1) samples came from terrestrial biomes of soil, dead or live plant material (e.g., soil, litter, rhizosphere soil, topsoil, lichen, deadwood, root, and shoot) and were not subject to experimental treatment that artificially modifies the fungal community composition (e.g., temperature or nitrogen increase experiment, greenhouse controlled experiment were excluded); (2) the precise geographic location of each sample was recorded and released using GPS coordinates; (3) the whole fungal community was subject to amplicon sequencing (studies using group-specific primers were excluded); (4) the internal transcribed spacer regions (ITS1, ITS2, or both) were subject to amplification; (5) sequencing data (either in fasta with phred scores reported or fastq format) were publicly available or provided by the authors of the study upon request, and the sequences were unambiguously assigned to samples; (6) the samples could be assigned to biomes according to the Environment Ontology (http://www.ontobee.org/ontology/ENVO)^8^. In total, 178 publications contained samples that matched our criteria.

### Processing of sequencing data

**Fig. 2:**
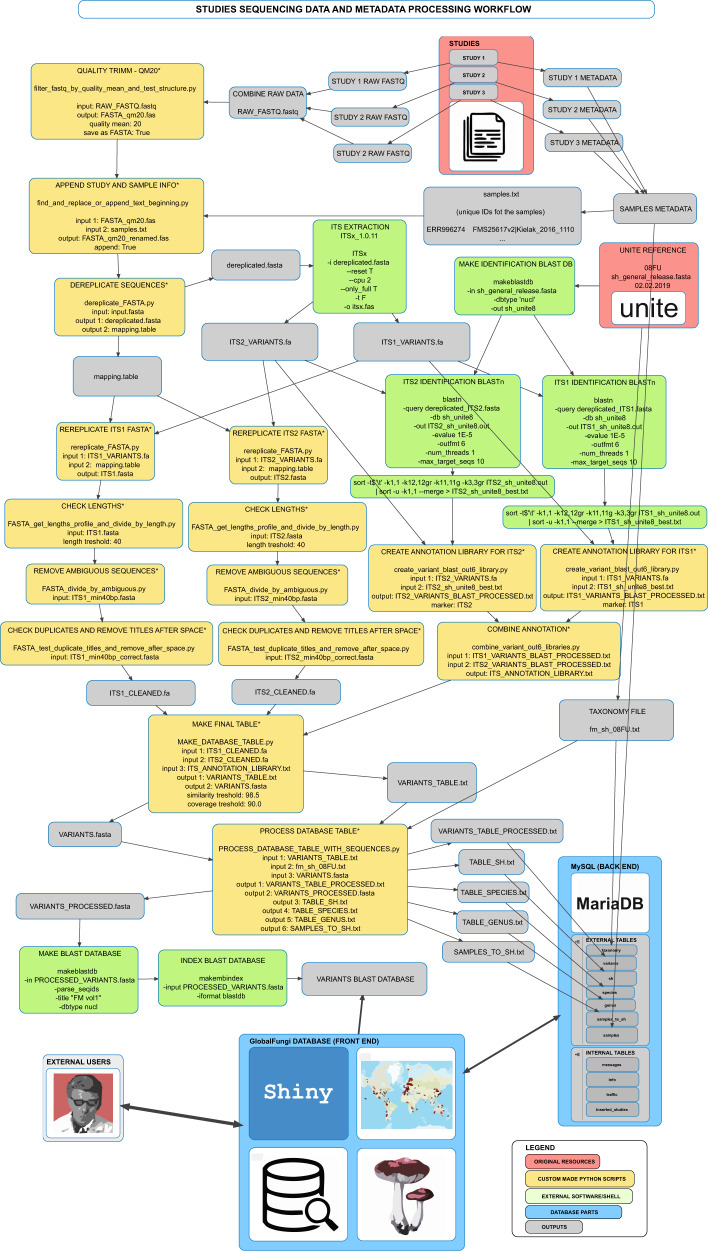
Processing of raw sequencing data for the GlobalFungi database. Workflow of processing of sequencing data included in the GlobalFungi database.

For the processing of data, see Fig. 2 and Code Availability section. Raw datasets from 178 studies, covering 17 242 individual samples were quality filtered by removing all sequences with the mean quality phred scores below 20. Each sequence was labelled using the combination of a sample ID and sequence ID, and the full ITS1 or ITS2 fungal region was extracted using Perl script ITSx v1.0.11^13^. ITS extraction resulted in a total of 416 291 533 full ITS1 and 231 278 756 full ITS2 sequences. The extracted ITS sequences were classified according to the representative sequence of the closest UNITE species hypothesis (SH) using BLASTn^14^, using the SH created considering a 98.5% similarity threshold (BLASTDBv5, general release 8.1 from 2.2.2019^12^). A sequence was classified to the best best hit SH only when the following thresholds were met: e-value < 10e^−50^, sequence similarity >  = 98.5%. All representative sequences annotated as nonfungal were discarded. All representative sequences classified to any fungal SH and all unclassified sequences were used to build database library of unique nucleotide sequences (sequence variants). The number of sequence variants accessible through the database is 113 423 871.

### Sample metadata

Sample metadata were collected from the published papers and/or public repositories where they were submitted by the authors. In some cases, metadata were obtained from the authors of individual studies upon request. The samples were assigned to continents, countries, and specific locations when available, and all sites were categorized into biomes following the classification of Environment Ontology to a maximum achievable depth for each sample. The complete list of metadata included in the database is presented in Table 1.

**Table 1:** List of metadata contained in the GlobalFungi database.

Metadata identifier	Unit	Description of content	Source
Sample ID		unique identifier	generated
Longitude	degrees	Geographical longitude	original paper
Latitude	degrees	Geographical latitude	original paper
Continent		One of the following: Africa/Antarctica/Asia/Australia/Europe/North America/South America	original paper
Sample type		One of the following: soil/rhizosphere soil/litter/litter + humus/deadwood/lichen/shoot/root	original paper
Biome		One of the following: forest biome/woodland biome/shrubland biome/grassland biome/desert biome/tundra biome/mangrove biome/anthropogenic terrestrial biome/marine biome/freshwater biome/polar desert biome	original paper
Sampling year		Year of sample collection	original paper
Primers		Primers used	original paper
pH		pH	original paper
ITS total		Number of full ITS sequences extracted	generated
MAT (°C)	°C	Mean annual temperature from CHELSA database	CHELSA
MAP (mm)	mm	Mean annual precipitation from CHELSA database	CHELSA

The table lists identifiers, units and sources of metadata contained in the database with the description of their content. The data source “original paper” may also represent additional metadata provided by the authors of the paper.

In addition to the metadata provided by the authors of each study, we also extracted bioclimatic variables from the global CHELSA^15^ and WorldClim 2^16^ databases for each sample based on its GPS location. Since the results based on CHELSA and WorldClim 2 were comparable, we decided to include those from CHELSA, because precipitation patterns are better captured in the CHELSA dataset, in particular for mountain sites^15^.

For each sequence variant that was classified to SH, fungal species name and genus name was retrieved from the UNITE database^12^, when available.

## Data Records

The raw sequencing reads used to create the database are available at different locations (see Table 2).

**Table 2:** List of identifiers and source database of the raw sequencing datasets used.

Database	Accession Identifiers (in superscripts, respectively: dataset reference, study reference(s))
National Center for Biotechnology Information Sequence Read Archive	SRP001058^19,20^ SRP001175^21,22^, SRP006078^23,24^, SRP012868^25,26^, SRP013695^27,28^, SRP013944^29,30^, SRP015735^31,32^, SRP016090^33,34^, SRP026207^35,36^, SRP028404^37,38^, SRP033719^39,40^, SRP035356^41,42^, SRP040314^43,44^, SRP040786^45,46^, SRP041347^47,48^, SRP043106^49,50^, SRP043706^4,51–53^, SRP043982^54,55^, SRP044665^56,57^, SRP045166^58,59^, SRP045587^60,61^, SRP045746^62,63^, SRP045933^64,65^, SRP046049^66,67^, SRP048036^68–70^, SRP048856^71,72^, SRP049544^73,74^, SRP051033^75,76^, SRP052222^77,78^, SRP052716^79,80^, SRP055957^81,82^, SRP057433^83,84^, SRP057541^85,86^, SRP058508^87,88^, SRP058555^89,90^, SRP058851^91,92^, SRP059280^93,94^, SRP060838^95,96^, SRP061179^97,98^, SRP061305^99,100^, SRP061904^101,102^, SRP062647^103,104^, SRP063711^105,106^, SRP064158^107,108^, SRP065817^109,110^, SRP066030^111,112^, SRP066284^113,114^, SRP066331^115,116^, SRP067301^117,118^, SRP067367^119,120^, SRP068514^121,122^, SRP068608^123,124^, SRP068620^125,126^, SRP068654^127,128^, SRP069065^129,130^, SRP069742^131,132^, SRP070568^133,134^, SRP073070^135,136^, SRP073265^137,138^, SRP074055^139,140^, SRP074496^141,142^, SRP075989^143,144^, SRP079403^145,146^, SRP079521^147,148^, SRP080210^149,150^, SRP080428^151,152^, SRP080680^153,154^, SRP082472^155,156^, SRP082976^157,158^, SRP083394^159,160^, SRP083434^160,161^, SRP083901^162,163^, SRP087715^164,165^, SRP090261^166,167^, SRP090335^168,169^, SRP090490^170,171^, SRP090651^172,173^, SRP091741^174,175^, SRP091855^176,177^, SRP091867^178,179^, SRP092609^180,181^, SRP092777^182,183^, SRP093592^184,185^, SRP093928^186,187^, SRP094708^188–190^, SRP097883^191,192^, SRP101553^193,194^, SRP101605^195,196^, SRP102378^197,198^, SRP102417^199,200^, SRP102775^201,202^, SRP106137^203,204^, SRP106774^205,206^, SRP107174^207,208^, SRP107743^209,210^, SRP109164^211,212^, SRP109773^213,214^, SRP110522^215,216^, SRP110810^217,218^, SRP113348^219,220^, SRP114697^221,222^, SRP114821^223,224^, SRP115350^225,226^, SRP115464^227,228^, SRP115599^229,230^, SRP117302^231,232^, SRP118875^233,234^, SRP118960^235,236^, SRP119174^237,238^, SRP125864^239,240^, SRP132277^241,242^, SRP132591^243,244^, SRP132598^244,245^, SRP136886^246,247^, SRP139483^248,249^, SRP142723^250,251^, SRP148813^252,253^, SRP150527^254,255^, SRP151262^256,257^, SRP153934^258,259^, SRP160913^260,261^, SRP161632^262,263^, SRP195764^264,265^
European Nucleotide Archive Sequence Read Archive	ERP001713^266,267^, ERP003251^268,269^, ERP003790^270,271^, ERP005177^272,273^, ERP005905^274,275^, ERP009341^276,277^, ERP010027^278,279^, ERP010084^280,281^, ERP010743^282,283^, ERP011924^284,285^, ERP012017^286,287^, ERP013208^288,289^, ERP013987^290,291^, ERP014227^292,293^, ERP017480^294,295^, ERP017851^296,297^, ERP017915^298,299^, ERP019580^300,301^, ERP019924^302,303^, ERP020657^304,305^, ERP022511^306,307^, ERP022742^308,309^, ERP023275^310,311^, ERP023718^312,313^, ERP023855^314,315^, ERP106131^316,317^, ERP107634^318,319^, ERP107636^319,320^, ERP110188^321,322^, ERP112007^323,324^
DNA Data Bank of Japan	DRA000926^325,326^, DRA000937^327,328^, DRA001737^329,330^, DRA002424^331,332^, DRA002469^333,334^, DRA003024^335,336^, DRA003730^337,338^, DRA004913^339,340^, DRA006519^341,342^, DRP002783^343,344^, DRP003138^345,346^, DRP005365^347,348^
Dryad Digital Repository	10.5061/dryad.2fc32^349,350^, 10.5061/dryad.n82g9^351,352^, 10.5061/dryad.2343k^353,354^, 10.5061/dryad.gp302^355,356^, 10.5061/dryad.cq2rb^357,358^, 10.5061/dryad.8fn8j^359,360^, 10.5061/dryad.216tp^361,362^
GenBank	KAYV00000000.1^363,364^, KAYU00000000.1^364,365^, KAYT00000000.1^364,366^, SAMN02934078^367,368^, SAMN02934079^368,369^
Australian Antarctic Data Center database	10.4225/15/526f42ada05b1^370,371^
Supplemental Data	Hartmann et al. (2012)^Supplementary_Data2,^^372^, Rime et al. (2016)^Fungi_SeqsID,^^373^

The database contains two data types: sequence variants (individual nucleotide sequences) and samples. For each sequence variant, the following information is stored: sequence variant code, identification of samples where sequence variant occurs and the number of observations, the SH of best hit (when available), fungal species name (when available), fungal genus name (when available). For each sample, metadata information is stored (Table 1). Sequence data and metadata are accessible at Figshare^17^ (GlobalFungi_ITS_variants.zip, GlobalFungi_metadata.xlsx). All database content is accessible using a public graphical user interface at https://globalfungi.com.

## Technical Validation

The technical validation included the screening of the data sources, sequencing data and data reliability. Regarding the data source screening, the data sources (published papers) were screened to fulfil the criteria outlined in the Methods section. The dataset was thoroughly checked for duplicates, and for all records that appeared in multiple publications, only the first original publication of the dataset was considered as a data source. Considering sequence quality, we have only utilized those primer pairs that are generally accepted to target general fungi (see Online-Only Table 1)^7,18^. Sequences were quality filtered by removing all sequences with the mean quality phred scores below 20 and sequences that did not represent complete ITS1 or ITS2 after extraction or those that were identified as chimeric by the ITS extraction software^13^ were removed. All representative sequences where the BLASTn search against the UNITE database^12^ resulted in a nonfungal organism, were discarded.

For data reliability, the geographic location represented by the GPS coordinates was validated first. For each sample set, the geographic location of the sample described in the text of the study was confronted with the location on the map. For those samples where disagreement was recorded (e.g., terrestrial samples positioned in the ocean or located in another region than described in the text), the authors of each study were asked for correction. Studies or samples that could not be reconciled in this way were excluded from the database. The quality of sample metadata was checked and if they were outside the acceptable range (such as content of elements or organic matter > 100%), these invalid metadata were removed.

## Usage Notes

**Fig. 3:**
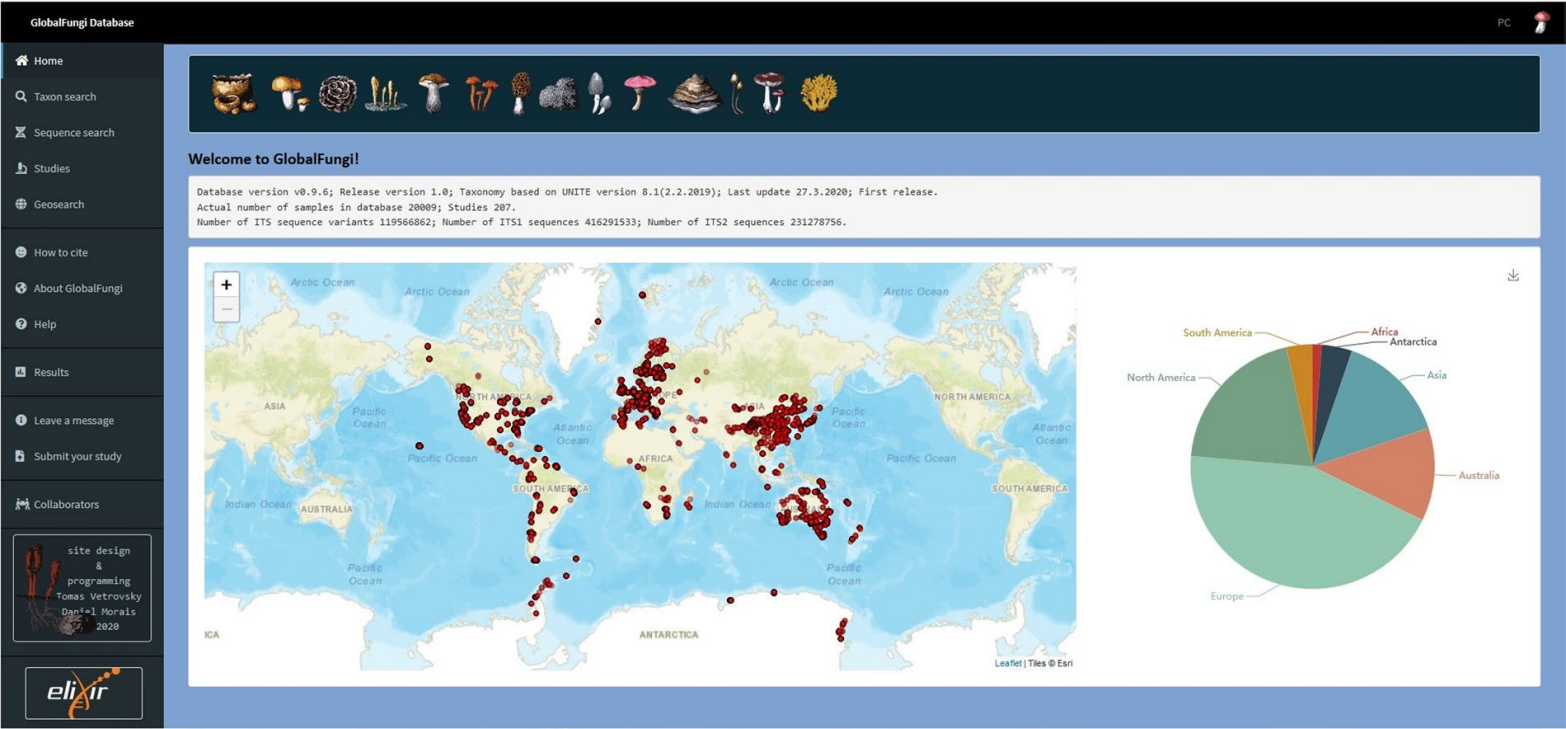
User interface to access the GlobalFungi database.

The user interface at https://globalfungi.com enables the users to access the database in several ways (Fig. 3). In the taxon search, it is possible to search for genera or species of fungi or for the 98.5% SH species hypotheses of UNITE, contained in the general release 8.1 from 2.2.2019. The search results open the options to download the corresponding SH or the corresponding sequence variants. It is also possible to view a breakdown of samples by type, biome, mean annual temperature, mean annual precipitation, pH, and continents. The results also contain an interactive map of the taxon distribution with relative abundances of sequences of the taxon across samples and a list of samples with metadata. Several modes of filtering of results are available as well.

In the sequence search, it is possible to search for multiple nucleotide sequences by choosing if the result will be the exact match or a BLAST result. The BLAST option gives the possibility to retrieve the sequence variant best hit in the database, or, when only one sequence is submitted, it is possible to display multiple ranked high score hits among the sequence variants.

It is also possible to open individual studies and access their content. Finally, in the Geosearch, users can select a group of samples on the map, with a range of tools, and retrieve data for these samples (such as the FASTA file with all occurring sequence variants).

Importantly, the database is intended to grow, both by the continuing activity of the authors and by using the help of the scientific community. For that, the “Submit your study” section of the web interface enabling the submission of studies not yet represented is available to users. The submission tool guides the submitting person through the steps where details about the publication, samples, sample metadata and sequences are sequentially submitted. The submitted data will be used to update the database twice a year after processing and validation by the authors. Thus, users submitting their data, besides a precious contribution to mycological progress, will benefit from making their data accessible to the international scientific community in an easily accessible form and increasing the visibility of their results. Users can also maximize their visibility by approving to add their name and affiliation to the online list of collaborators and/or to the GlobalFungi Group Author’ list that will be mentioned in future publications describing the database content, its development, or metastudies using the whole database.

Among the possible uses of the GlobalFungi Database, fungal ecologists will be able to link fungal diversity data with the panel of collected metadata, which should allow them to determine the environmental factors driving the fungal diversity. This kind of study can be done at different geographic levels, from country scale up to the entire world, and for all the fungal communities or by focusing on some ecosystem compartments. This should lead to a better understanding of the biogeography of the fungal diversity. Větrovský *et al*.^8^ brought interesting findings by doing this for soil fungal communities at the scale of the globe. The evolutionary biologists could study, for example, the effect of global change on the fungal diversity by comparing the natural versus anthropogenic biomes. In addition to focus on the fungal diversity, some studies could trigger specific fungi. Thus, mycologists could determine the biogeography of one specific fungal species. They could also determine the composition of the fungal communities associated with the focused species and detect some potential recurrent fungal associations. The GlobalFungi Database could also speed up the progress in fungal taxonomy by highlighting the existence of a high number of fungal sequences not currently assigned to species along with environmental metadata promoting thus the interest in their description.

## Data Availability

The workflow included several custom made python scripts (labelled by star in the Fig. 2) which are accessible here: https://github.com/VetrovskyTomas/GlobalFungi.

## Online-only Table

**Online-only Table 1:** List of primer pairs to amplify general fungal communities in the samples included in this Data Descriptor^7,17^.

Primer pair	Sequence
1737F / 2043 R	GGAAGTAAAAGTCGTAACAAGG / GCTGCGTTCTTCATCGATGC
18S-F / 5.3S-R	GTAAAAGTCGTAACAAGGTTTC / GTTCAAAGAYTCGATGATTCAC
58A2F / ITS4	ATCGATGAAGAACGCAG / TCCTCCGCTTATTGATATGC
fITS7 / ITS4	GTGARTCATCGAATCTTTG / TCCTCCGCTTATTGATATGC
fITS9 / ITS4	GAACACAGCGAAATGTGA / TCCTCCGCTTATTGATATGC
gITS7 / ITS4	GTGARTCATCGARTCTTTG / TCCTCCGCTTATTGATATGC
gITS7 / ITS4ngs	GTGARTCATCGARTCTTTG / TTCCTSCGCTTATTGATATGC
ITS1 / ITS2	TCCGTAGGTGAACCTGCGG / GCTGCGTTCTTCATCGATGC
ITS1 / ITS4	TCCGTAGGTGAACCTGCGG / TCCTCCGCTTATTGATATGC
ITS1F / 58A2R	CTTGGTCATTTAGAGGAAGTAA / CTGCGTTCTTCATCGAT
ITS1F / gITS7	CTTGGTCATTTAGAGGAAGTAA / GTGARTCATCGARTCTTTG
ITS1F / ITS2	CTTGGTCATTTAGAGGAAGTAA / GCTGCGTTCTTCATCGATGC
ITS1F / ITS3R	CTTGGTCATTTAGAGGAAGTAA / TCCTCCGCTTATTGATATGC
ITS1F / ITS4	CTTGGTCATTTAGAGGAAGTAA / TCCTCCGCTTATTGATATGC
ITS1F_KYO1 / ITS2_KYO1	CTHGGTCATTTAGAGGAASTAA / CTRYGTTCTTCATCGDT
ITS1F_KYO1 / ITS2_KYO2	CTHGGTCATTTAGAGGAASTAA / TTYRCTRCGTTCTTCATC
ITS1F_KYO2 / ITS2_KYO2	TAGAGGAAGTAAAAGTCGTAA / TTYRCTRCGTTCTTCATC
ITS1F_KYO2 / LR3	TAGAGGAAGTAAAAGTCGTAA / CCGTGTTTCAAGACGGG
ITS1FI2 / 5.8 S	GAACCWGCGGARGGATCA / CGCTGCGTTCTTCATCG
ITS1FI2 / ITS2	GAACCWGCGGARGGATCA / GCTGCGTTCTTCATCGATGC
ITS1Fngs / ITS2	GGTCATTTAGAGGAAGTAA / GCTGCGTTCTTCATCGATGC
ITS1ngs / ITS2	TCCGTAGGTGAACCTGC / GCTGCGTTCTTCATCGATGC
ITS1-OF / ITS4	AACT(C)GG(C)CATTTAGAGGAAGT / TCCTCCGCTTATTGATATGC
ITS2 / ITS4	GCTGCGTTCTTCATCGATGC / TCCTCCGCTTATTGATATGC
ITS2 / ITS5	GCTGCGTTCTTCATCGATGC / GGAAGTAAAAGTCGTAACAAGG
ITS2F / ITS2R	GCATCGATGAAGAACGC / CCTCCGCTTATTGATATGC
ITS3 / ITS4	GCATCGATGAAGAACGCAGC / TCCTCCGCTTATTGATATGC
ITS3_KYO2 / ITS4	GATGAAGAACGYAGYRAA / TCCTCCGCTTATTGATATGC
ITS3_KYO2 / ITS4F	GATGAAGAACGYAGYRAA / CGCTTATTRATATGCTTAAXGXT
ITS3_KYO2 / LR_KYO1b	GATGAAGAACGYAGYRAA / MGCWGCATTCCCAAACWA
ITS3ngs mix / ITS4ngr mix + ARCH-ITS4-F1	CA(T)CGATGAAGAACG(C)(A)G / TCCT(S)(C)(G)CTTATTGATATGC
ITS3ngs1 to ITS3ngs11 / ITS4ngs	(C)(A)(T)CGATGAAGA(A)CG(C)(A)G / TCCTSCGCTTATTGATATGC
ITS3tagmix1 to ITS3tagmix5 / ITS4ngs	CTAGACTCGTCA(T)CGATGAAGAACG(CA)G / TTCCTSCGCTTATTGATATGC
ITS4 / fITS9	TCCTCCGCTTATTGATATGC / GAACACAGCGAAATGTGA
ITS4 / ITS3_KYO2	TCCTCCGCTTATTGATATGC / GATGAAGAACGYAGYRAA
ITS4 / ITS5	TCCTCCGCTTATTGATATGC / GGAAGTAAAAGTCGTAACAAGG
ITS4 / ITS9	TCCTCCGCTTATTGATATGC / GCATTAGAAACTGCTCGTAATG
ITS5 / 5.8S_fungi	GGAAGTAAAAGTCGTAACAAGG / CAAGAGATCCGTTGTTGAAAGTT
ITS5 / ITS2	GGAAGTAAAAGTCGTAACAAGG / GCTGCGTTCTTCATCGATGC
ITS5 / ITS2_KYO2	GGAAGTAAAAGTCGTAACAAGG / TTYRCTRCGTTCTTCATC
ITS5 / ITS4	GGAAGTAAAAGTCGTAACAAGG / TCCTCCGCTTATTGATATGC
ITS7o / ITS4	GTGAATCATCRAATYTTTG / TCCTCCGCTTATTGATATGC
ITS86F / ITS4	GTGAATCATCGAATCTTTGAA / TCCTCCGCTTATTGATATGC
ITS9 / ITS4	GCATTAGAAACTGCTCGTAATG / TCCTCCGCTTATTGATATGC
ITS9F / ITS4	CCATCTCATCCCTGCGTGTCTCCGACTCAG / TCCTCCGCTTATTGATATGC
