# Author Correction: GlobalFungi, a global database of fungal occurrences from high-throughput-sequencing metabarcoding studies

**DOI:** 10.1038/s41597-020-00647-3

**Published:** 2020-09-15

**Authors:** Tomáš Větrovský, Daniel Morais, Petr Kohout, Clémentine Lepinay, Camelia Algora, Sandra Awokunle Hollá, Barbara Doreen Bahnmann, Květa Bílohnědá, Vendula Brabcová, Federica D’Alò, Zander Rainier Human, Mayuko Jomura, Miroslav Kolařík, Jana Kvasničková, Salvador Lladó, Rubén López-Mondéjar, Tijana Martinović, Tereza Mašínová, Lenka Meszárošová, Lenka Michalčíková, Tereza Michalová, Sunil Mundra, Diana Navrátilová, Iñaki Odriozola, Sarah Piché-Choquette, Martina Štursová, Karel Švec, Vojtěch Tláskal, Michaela Urbanová, Lukáš Vlk, Jana Voříšková, Lucia Žifčáková, Petr Baldrian

**Affiliations:** 1 Institute of Microbiology of the Czech Academy of Sciences, Vídeňská 1083, 14220 Praha 4, Czech Republic; 2 Laboratory of Systematic Botany and Mycology, University of Tuscia, Largo dell’Università snc, Viterbo, 01100 Italy; 3 Department of Forest Science and Resources, College of Bioresource Sciences, Nihon University, Fujisawa, Kanagawa Japan; 4 Department of Biology, United Arab emirates University, Al Ain, Abu Dhabi United Arab Emirates; 5 Section for Genetics and Evolutionary Biology, University of Oslo, Blindernveien 31, 0316 Oslo, Norway

Correction to: *Scientific Data* 10.1038/s41597-020-0567-7, published online 13 July 2020

The legend of Fig. 1 has been updated to include the following text acknowledging the source and copyright of the background map image:

“The background map image where the samples are represented is the intellectual property of Esri and used herein under license. Copyright © 2019 Esri and its licensors. All rights reserved.”

